# Supplementary material for: Diagnostic limitations of clinical case definitions of pertussis in infants and children with severe lower respiratory tract infection
Source: PLoS One. 2020 Jul 17;15(7):e0235703. doi: 10.1371/journal.pone.0235703 (PMC7367487; doi:10.1371/journal.pone.0235703)
Supplement: S1 Data — (PDF) [file pone.0235703.s001.pdf]

Minimal dataset

Fig 1. Sensitivity and specificity of clinical features in the diagnosis of pertussis

A: Wold Health Organization

	**Clinical Feature**	PCR +	PCR -
		n=13	n =119
		n; % (95 CI)	n; % (95 CI)
	Paroxysmal cough	8; 62% (34 - 83)	40; 34% (26 - 43)
	Whoop	4; 31% (12 - 59)	82; 69% (60 - 77)
**0 - 3 months**	Post-tussive vomit	7; 54% (28 - 78)	63; 53% (44 - 62)
	Any	9; 69% (41 - 88)	23; 19% (13 - 27)
		n=19	n =307
	Paroxysmal cough	15; 79% (55 - 92)	89; 29% (24 - 34)
	Whoop	4; 76% (71 - 81)	234; 76% (71 - 81)
**4 months - 9 years**	Post-tussive vomit	5; 26% (11 - 50)	177; 58% (52 - 63)
	Any	16; 84% (61 - 95)	43; 14% (11 - 18)

B: Global Pertussis Initiative

	**Clinical Feature**	PCR +	PCR -
		n=13	n =119
		n; % (95 CI)	n; % (95 CI)
	Whoop	4; 31% (12 - 59)	86; 72% (63 - 80)
	Apnea	2; 15% (4 - 45)	112; 94% (88 - 97)
	Post-tussive vomit	7; 54% (28 - 78)	70; 59% (50 - 67)
**0 - 3 months**	Cyanosis	2; 15% (4 - 45)	117; 98% (93 - 100)
	Any	7; 54% (28 - 78)	57; 48% (39 - 57)
		n=19	n =307
		n; % (95 CI)	n; % (95 CI)
	Whoop	2; 11% (3 - 34)	269; 88% (83 - 91)
	Apnea	1; 5% (1 - 30)	301; 98% (96 - 99)
**4 months - 9 years**	Post-tussive vomit	3; 16% (5 - 39)	240; 78% (73 - 82)
	Cyanosis	1; 5% (1 - 30)	296; 96% (94 - 98)
	Any	4; 21% (8 - 45)	219; 71% (66 - 76)

95% CI = 95% Confidence Interval

Fig 2 & Fig 3

A World Health Organization

1. Detailed report of sensitivity and specificity for the whole group

Cutpoint	Sensitivity	Specificity	Correctly Classified	LR+	LR-
(>= 1)	100.00%	0.00%	6.51%	1.0000	
(>= 2)	100.00%	5.57%	11.72%	1.0590	0.0000
(>= 3)	72.00%	34.26%	36.72%	1.0953	0.8172
(>= 4)	56.00%	57.38%	57.29%	1.3140	0.7668
(>= 5)	40.00%	69.64%	67.71%	1.3174	0.8616
(>= 6)	32.00%	79.11%	76.04%	1.5317	0.8596
(>= 7)	32.00%	81.89%	78.65%	1.7674	0.8303
(>= 8)	12.00%	92.76%	87.50%	1.6569	0.9487
(>= 9)	12.00%	93.59%	88.28%	1.8730	0.9402
(>= 10)	12.00%	93.87%	88.54%	1.9582	0.9374
(>= 14)	12.00%	94.43%	89.06%	2.1540	0.9319

Obs	ROC Area	Std. Err.	Asymptotic Normal [95% Conf.	Interval]
384	0.5815	0.0599	0.46405	0.69896

2. Detailed report of sensitivity and specificity: 1 - 3 months old

Cutpoint	Sensitivity	Specificity	Correctly Classified	LR+	LR-
(>= 1)	100.00%	0.00%	8.57%	1.0000	
(>= 2)	100.00%	4.17%	12.38%	1.0435	0.0000
(>= 3)	88.89%	25.00%	30.48%	1.1852	0.4444
(>= 4)	77.78%	52.08%	54.29%	1.6232	0.4267
(>= 5)	66.67%	62.50%	62.86%	1.7778	0.5333
(>= 6)	55.56%	75.00%	73.33%	2.2222	0.5926
(>= 7)	55.56%	79.17%	77.14%	2.6667	0.5614
(>= 8)	33.33%	92.71%	87.62%	4.5714	0.7191
(>= 14)	33.33%	93.75%	88.57%	5.3333	0.7111

Obs	ROC Area	Std. Err.	Asymptotic Normal [95% Conf.	Interval]
105	0.7130	0.1005	0.51604	0.90989

3. Detailed report of sensitivity and specificity: 4 months to 9 years of age

Cutpoint	Sensitivity	Specificity	Correctly Classified	LR+	LR-
(>= 1)	100.00%	0.00%	5.73%	1.0000	
(>= 2)	100.00%	6.08%	11.47%	1.0648	0.0000
(>= 3)	62.50%	37.64%	39.07%	1.0023	0.9962
(>= 4)	43.75%	59.32%	58.42%	1.0754	0.9483
(>= 5)	25.00%	72.24%	69.53%	0.9007	1.0382
(>= 6)	18.75%	80.61%	77.06%	0.9669	1.0080
(>= 7)	18.75%	82.89%	79.21%	1.0958	0.9802
(>= 8)	0.00%	92.78%	87.46%	0.0000	1.0779
(>= 9)	0.00%	93.54%	88.17%	0.0000	1.0691
(>= 10)	0.00%	93.92%	88.53%	0.0000	1.0648
(>= 14)	0.00%	94.68%	89.25%	0.0000	1.0562

Obs	ROC Area	Std. Err.	Asymptotic Normal [95% Conf. Interval]	
279	0.5087	0.0687	0.37400	0.64335

B .Global Pertussis Initiative

1. Detailed report of sensitivity and specificity: whole group

Cutpoint	Sensitivity	Specificity	Correctly Classified	LR+	LR-
(>= 1)	100.00%	0.00%	6.83%	1.0000	
(>= 2)	100.00%	2.67%	9.32%	1.0274	0.0000
(>= 3)	90.91%	28.00%	32.30%	1.2626	0.3247
(>= 4)	81.82%	50.00%	52.17%	1.6364	0.3636
(>= 5)	72.73%	62.00%	62.73%	1.9139	0.4399
(>= 6)	63.64%	72.67%	72.05%	2.3282	0.5004
(>= 7)	63.64%	76.67%	75.78%	2.7273	0.4743
(>= 8)	27.27%	87.33%	83.23%	2.1531	0.8328
(>= 9)	27.27%	88.67%	84.47%	2.4064	0.8202
(>= 10)	27.27%	89.33%	85.09%	2.5568	0.8141
(>= 14)	27.27%	90.67%	86.34%	2.9221	0.8021

Obs	ROC Area	Std. Err.	Asymptotic Normal [95% Conf. Interval]	
161	0.7197	0.0804	0.56214	0.87725

2. Detailed report of sensitivity and specificity: 1 - 3 months

Cutpoint	Sensitivity	Specificity	Correctly Classified	LR+	LR-
(>= 1)	100.00%	0.00%	10.14%	1.0000	
(>= 2)	100.00%	1.61%	11.59%	1.0164	0.0000
(>= 3)	85.71%	11.29%	18.84%	0.9662	1.2653
(>= 4)	85.71%	38.71%	43.48%	1.3985	0.3690
(>= 5)	85.71%	53.23%	56.52%	1.8325	0.2684
(>= 6)	71.43%	72.58%	72.46%	2.6050	0.3937
(>= 7)	71.43%	77.42%	76.81%	3.1633	0.3690
(>= 8)	42.86%	88.71%	84.06%	3.7959	0.6442
(>= 14)	42.86%	90.32%	85.51%	4.4286	0.6327

Obs	ROC Area	Std. Err.	Asymptotic Normal [95% Conf.	Interval]
69	0.7442	0.1242	0.50073	0.98775

3. Detailed report of sensitivity and specificity: 4 months to 9 years

Cutpoint	Sensitivity	Specificity	Correctly Classified	LR+	LR-
(>= 1)	100.00%	0.00%	4.35%	1.0000	
(>= 2)	100.00%	3.41%	7.61%	1.0353	0.0000
(>= 3)	100.00%	39.77%	42.39%	1.6604	0.0000
(>= 4)	75.00%	57.95%	58.70%	1.7838	0.4314
(>= 5)	50.00%	68.18%	67.39%	1.5714	0.7333
(>= 6)	50.00%	72.73%	71.74%	1.8333	0.6875
(>= 7)	50.00%	76.14%	75.00%	2.0952	0.6567
(>= 8)	0.00%	86.36%	82.61%	0.0000	1.1579
(>= 9)	0.00%	87.50%	83.70%	0.0000	1.1429
(>= 10)	0.00%	88.64%	84.78%	0.0000	1.1282
(>= 14)	0.00%	90.91%	86.96%	0.0000	1.1000

Obs	ROC Area	Std. Err.	Asymptotic Normal [95% Conf.	Interval]
92	0.6861	0.0877	0.51420	0.85796

Fig 4. Sensitivity and specificity of lymphocytosis in the diagnosis of pertussis with and without World Health Organization and Global Pertussis Initiative criteria

		PCR +	PCR -
		n/n; % (95 CI)	n/n; % (95 CI)
**0 - 3 months**		n=13	n =119
	Lymphocytosis alone	4/13; 31% (12 – 59)	90/119; 76% (67 - 83)
	+ World Health Organization	4/9; 44% (17 – 75)	18/23; 78% (56 - 91)
	+ Global Pertussis Initiative	4/7; 57% (23 - 86)	44/57; 77% (64 - 86)
**4 months - 9 years**		n=19	n =307
	Lymphocytosis alone	6/19; 32% (15 – 55)	211/307; 69% (63 - 74)
	+ World Health Organization	5/16; 31% (14 – 57)	32/43; 74% (59 - 85)
	+ Global Pertussis Initiative	1/4; 25% (3 - 77)	153/219; 70% (63 - 76)
**Whole group (all)**		n=32	n =426
	Lymphocytosis alone	10/32; 31% (18 - 49)	301/426; 71% (66 - 75)
	+ World Health Organization	9/25; 36% (20 – 56)	50/66; 76% (64 - 85)
	+ Global Pertussis Initiative	5/11; 45% (20 – 73)	197/276; 71% (66 – 76)

95% CI = 95% Confidence Interval
